# Nutrient vitamins enabled metabolic regulation of ferroptosis via reactive oxygen species biology

**DOI:** 10.3389/fphar.2024.1434088

**Published:** 2024-07-18

**Authors:** Junjie Wu, Yanting Shi, Man Zhou, Min Chen, Shuying Ji, Xingxing Liu, Mengjiao Zhou, Rui Xia, Xiaohua Zheng, Weiqi Wang

**Affiliations:** ^1^ School of Pharmacy, Nantong University, Nantong, Jiangsu, China; ^2^ School of Public Health, Nantong University, Nantong, Jiangsu, China

**Keywords:** ferroptosis, vitamins, nutrient signaling, metabolic regulation, reactive oxygen species

## Abstract

Vitamins are dietary components necessary for cellular metabolic balance, especially redox homeostasis; deficient or excessive supply may give rise to symptoms of psychiatric disorders. Exploring the nutritional and metabolic pathways of vitamins could contribute to uncovering the underlying pathogenesis of ferroptosis-associated diseases. This mini-review aims to provide insights into vitamins closely linked to the regulation of ferroptosis from the perspective of cellular reactive oxygen species biology. The mainstream reprogramming mechanisms of ferroptosis are overviewed, focusing on unique biological processes of iron metabolism, lipid metabolism, and amino acid metabolism. Moreover, recent breakthroughs in therapeutic interventions targeting ferroptosis via fully utilizing vitamin-based pharmacological tools were overviewed, covering vitamins (B, C, E, and K). Finally, mechanism insight related to vitamin-associated nutrient signaling was provided, highlighting the pharmacological benefits of metabolically reprogramming ferroptosis-associated diseases.

## Introduction

Cell death can occur accidentally or controllably to maintain biological homeostasis by the removal of unnecessary cells and cell debris. In particular, regulated cell death controlled by interconnected signaling pathways is linked with tumorigenesis, apparently different from accidental cell death (Tang et al., 2019; Tong et al., 2022). Since its discovery in 1973, clinical oncology investigation has been devoted to promoting the development of therapeutics that are efficient in apoptosis induction (Kerr et al., 1972; Peng et al., 2022). In-depth studies of this lethal subroutine revealed the general characteristics of cancers that are resistant to apoptosis, and induction of other regulated routes involving necroptosis (Gong et al., 2019), autophagy (White, 2015), and pyroptosis (Yu et al., 2021), and so on. Over the past decades, regulated cell death pathways have been extensively studied in the field of cancer treatment, especially for newly emerging reactive oxygen species (ROS) nanomedicine that is amenable to the therapeutic intervention of ferroptosis execution. ROS-producing nanoparticles function by infiltrating cancerous tissues and exploiting the altered redox balance prevalent in these cells. Once activated, they intensify intracellular oxidative stress beyond the threshold cancer cells can manage, leading to uncontrolled lipid oxidation and membrane damage-hallmark of ferroptosis.

Up to 2012, ferroptosis featured phospholipid peroxidation driven by an iron-dependent manner, displaying significant differences at morphological, biochemically, and genetic levels (Dixon et al., 2012; Tang et al., 2020). Morphologically, unrepaired cellular damage spreads in a wave-like pattern, characterized by cytoplasmic and organelle ruptures, chromatin condensation, and a rise in autophagosomes (Riegman et al., 2020). Conceptually, ferroptosis was advanced to describe the result of iron accumulation and lipid peroxides (LPO) from the perspective of biochemistry (Liang et al., 2022). At present, GSH depletion and GPX4 inhibition play a crucial role in the initiation of ferroptosis, and other free radical-trapping targets involved ferroptosis suppressor protein 1 (FSP1) and GTP cyclohydrolase 1 (GCH1) (Jiang et al., 2021). To date, the underlying molecular mechanisms related to ferroptotic cell death have been extensively studied for therapeutic purposes.

Intriguingly, recent preclinical evidence suggests that certain malignancies are prone to chemoresistance and metastasis but are exquisitely vulnerable to ferroptosis (Liang et al., 2019; Mou et al., 2019; Lei et al., 2022). Consequently, modulating ferroptosis via metabolic interventions has garnered significant attention (Wu et al., 2020; Koppula et al., 2021). In this mini-review, we first summarized the key metabolic reprogramming of ferroptotic cell death, covering unique biological processes and pathophysiological characteristics in terms of iron metabolism, lipid metabolism, and amino acid metabolism. Further, we delve into the nutritional vitamins harnessed to govern ferroptosis, emphasizing their potential in tumor suppression and immune surveillance, underscoring the therapeutic promise of metabolic reprogramming in ferroptosis-linked diseases. Finally, we also outlined the current understanding of induction/inhibition mechanisms and conceptualized promising strategies that contribute to ROS biology for collaborative ferroptosis regulation.

## Nutrient vitamins assosiated ferroptosis regulation

**FIGURE 1:**
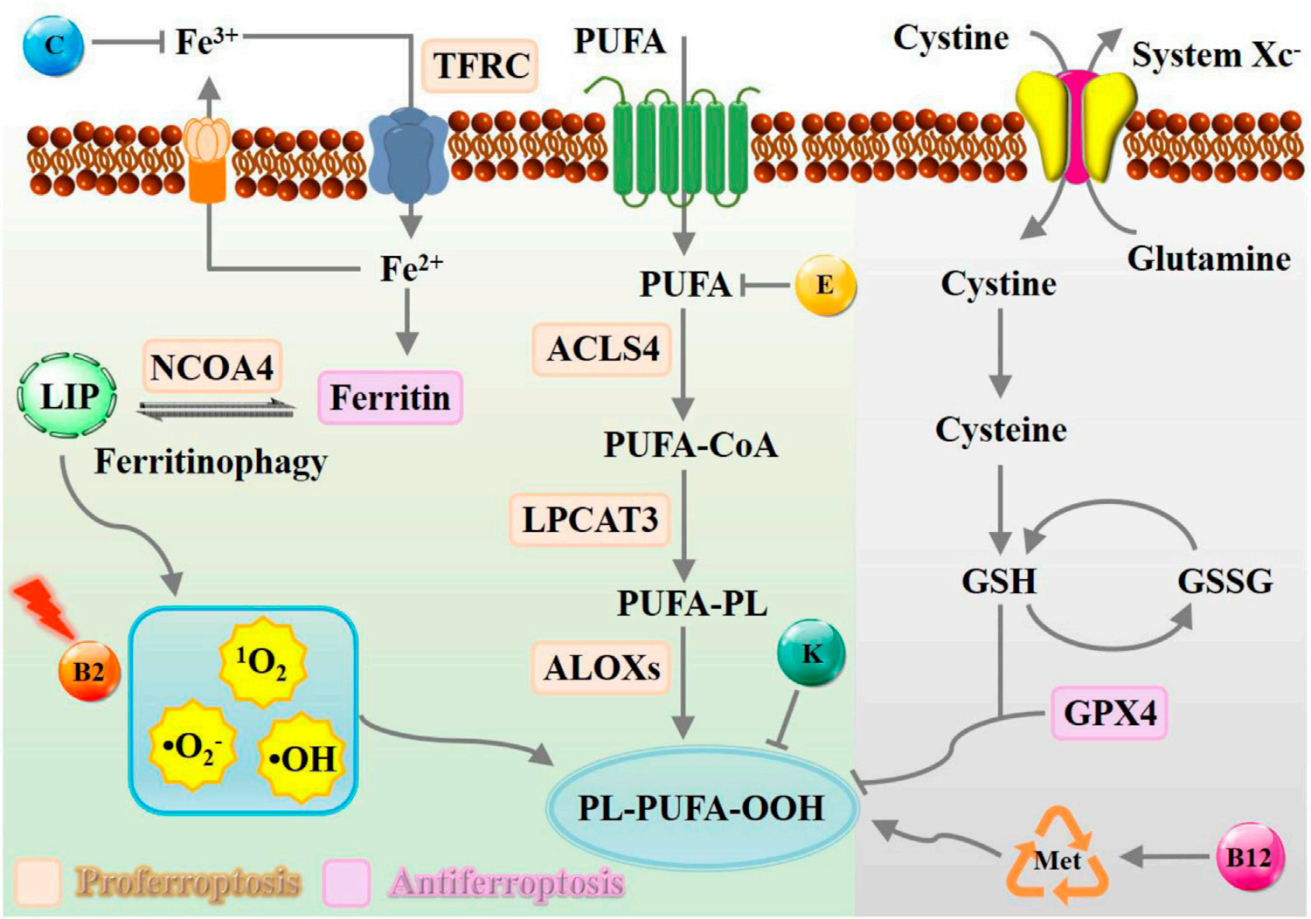
Schematic representation of metabolic processes of ferroptosis modulated by nutrient vitamins. The modulation of the ferroptosis process can be facilitated by the presence of vitamin B, C, E, and K, which exert a multifaceted influence towards iron deposition, ROS accumulation, and radical-trapping. Abbreviation: TFRC, transferrin receptor; NCOA4, nuclear receptor coactivator 4; LIP, labile iron pool; PUFA, polyunsaturated fatty acid; ACSL4, acyl-CoA synthetase long-chain family member 4; LPCAT3, lysophosphatidylcholine acyltransferase 3; LOX, lipoxygenase.

The downstream regulatory network of ferroptotic cell death is driven by the disequilibrium of redox hemostasis, owing to the inactivation of reducing enzymes or lipid oxidation products. As depicted in Figure 1, canonical nutrient vitamins actively participate in metabolic reprogramming mechanisms governing ferroptosis were outlined, including iron metabolism (Rizzollo et al., 2021), lipid metabolism (Snaebjornsson et al., 2020), and amino acid metabolism (Sies, 2021). Overall, vitamin B2 has been proven to be efficient in promoting ROS production assisted by laser or ultrasound irradiation, while B12 functions via methionine cycle to promote ferroptosis execution. In contrast, vitamin C may interfere in iron homeostasis, vitamin E act as endogenous antioxidants participate in neutralizing excessive free radicals to prevent oxidative damage of PUFA. Natural vitamin K is commonly recognized for its role as a potent inhibitor of ferroptosis, whereas synthetic variants can facilitate this process instead.

### Iron metabolism

Cancer cells exhibited strong iron dependency to support their rapid proliferation, which is usually accompanied by upregulated transferrin and downregulated ferroportin, thus can be harnessed to therapeutic interventions of ferroptosis-associated diseases (Bao et al., 2020; Bao et al., 2021). Excessive intracellular iron is accompanied by highly oxidative stress that can propagate LPO accumulation (Zhang S. et al., 2022; Zhu et al., 2022). Thus, sensitizing cancer cells to oxidative damage and ferroptosis can be orchestrated by modulating iron uptake, storage, utilization, and export (Hassannia et al., 2019). Generally, cellular transport systems function well to preserve iron homeostasis (Andrews and Schmidt, 2007; Bogdan et al., 2016). In brief, iron can be internalized by circulating glycoprotein transferrin and discharged by ferroportin (Zhang C. et al., 2020). The intracellular labile iron pool (LIP) maintains a delicate balance normally and can be elevated by transferrin-mediated uptake or autophagic degradation (Lin et al., 2020). Inhibition of nuclear receptor coactivator 4 (NCOA4) mediated ferritinophagic flux or abnormal expression of iron metabolism proteins iron-responsive element binding protein 2 can dramatically decrease the vulnerability to ferroptosis (Fang et al., 2021; Zhou H. et al., 2023). Especially, vitamin C may interference iron metabolism by enhancing iron absorption. In the biological system, iron is an essential micronutrient element that can be engaged in hydroxyl radicals (•OH) formation through the Haber-Weiss and Fenton reaction, relying on the cycle between the ferrous (Fe^2+^) and ferric (Fe^3+^) states (Wang et al., 2020). Vitamin C helps convert poorly absorbed Fe^3+^ into its more absorbable Fe^2+^. Additionally, vitamin C acts as an antioxidant, protecting against oxidative stress that are associated with iron metabolism, thereby maintaining iron homeostasis and overall metabolic health.

### Lipid metabolism

Lipid peroxidation is one of the canonical processes catalyzed by spontaneous autoxidation or endogenous enzymes that directly contribute to promoting ferroptosis (Yang and Stockwell, 2016; von Krusenstiern et al., 2023). At present, the widely recognized pathway is excessive peroxidation of polyunsaturated fatty acid (PUFA), comprising arachidonic acid and adrenic acid (Bazinet and Layé, 2014; Kagan et al., 2016). PUFAs, especially long-chain ones like ω-3 and ω-6 LCPUFAs, readily oxidize due to their numerous double bonds, rendering them susceptible to free radical assault. Vitamin E, an effective fat-loving antioxidant, acts as a membrane shield, with heightened importance in PUFA-dense areas. It directly neutralizes free radicals, halting their damaging cascade to preserve PUFAs. Teaming up with trace elements like selenium, it enhances the body’s antioxidant shield, defending both PUFAs and other cellular elements against oxidative stress. Additionally, vitamin E regulates metabolic interactions with PUFAs, impacting the progression of ferroptosis.

Generally, the peroxidation process of PUFA-containing phospholipids can be divided into three steps. Firstly, acyl-CoA synthetase long-chain family member 4 (ACSL4) converts the PUFA into acylated form (Gan, 2022). Then, lysophosphatidylcholine acyltransferase 3 (LPCAT3) participates in the process of esterifying acyl Co-A derivatives, incorporating PUFAs into phospholipid membranes (Hashidate-Yoshida et al., 2015). Finally, peroxidation occurs to form lipid hydroperoxide under the catalysis of lipoxygenase (LOX) (Stoyanovsky et al., 2019). Excessive oxidation of PUFA-containing phospholipids might disrupt the integrity of biological membranes, eventually decomposing into reactive toxic aldehydes (such as hydroxynonenal or malondialdehydes) facilitating ferroptosis execution (Dyall et al., 2022). Vitamin K1 has also emerged as a potent blocker of ferroptosis, a controlled cellular death mechanism linked to lipid oxidation. Research from Kolbrink et al. (Kolbrink et al., 2022) demonstrated its effectiveness in stopping ferroptosis in cellular models, reversing cell death and suggesting its potential in treating issues arising from oxygen deprivation and restoration cycles, like tissue injury. These discoveries emphasize vitamin K’s pivotal role, via unconventional metabolic pathways, in regulating ferroptosis-related diseases and its promise as a target for innovative therapies. Besides, the antioxidant mechanism of nutrient vitamins involves FSP1-triggered reduction, sustaining its radical-trapping properties to interrupt ferroptosis pathways.

### Amino acid metabolism

In the biological system, extracellular glutamate and cystine are transported into the cell by amino acid transporter receptors system Xc^−^, while inhibiting the function of system Xc^−^ can decrease the intracellular cysteine and GSH content (Koppula et al., 2021; Xiong et al., 2021). The cysteine required for GSH synthesis is generally obtained by the transsulfuration pathway of sulfur transfer from homocysteine to cysteine (Stockwell et al., 2020; Lee and Roh, 2022). GPX4 can protect cells from ferroptosis by reducing the lethal PUFA-containing phospholipid hydroperoxides (PL-OOH) into phospholipid alcohols (PL-OH) (Yang et al., 2014). Overall, the existing intracellular GSH/Xc^−^/GPX4 antioxidant defense system is of great importance in maintaining cellular redox homeostasis.

Moreover, the methionine-synthase cycle has been proven to be relevant to metabolic process of ROS generation, in which vitamin B12 plays a crucial role as a cofactor. It primarily functions to regenerate methionine, an essential amino acid, from homocysteine. The methionine cycle, involving SBP-1/SREBP1 and lipogenesis, is crucial for how early-life B12 levels influence later health outcomes. Research indicates that a lack of vitamin B12 early in life can result in elevated fat levels and toxic lipid accumulation, notably long-chain polyunsaturated fatty acids (LC-PUFAs), during adulthood, which triggers ferroptosis in reproductive cells (Qin et al., 2022). From the perspective of these signaling pathways, amino acid metabolism is crucial for the regulation of intracellular redox balance, especially amino acid-related metabolic processes, which could significantly affect the therapeutic effect of ferroptosis.

## Metabolic regulation of ferroptosis with vitamins

Multiple nutrients including exogenous fatty acids and amino acids have been reviewed to regulate redox homeostasis and ferroptosis execution (Koppula et al., 2018; Qi et al., 2021). However, the underlying mechanism of vitamins and ferroptosis and corresponding nutrient signaling related to ferroptosis regulation is still limited. Vitamins are dietary components that have functioned for therapeutic applications over the past decades (Saghiri et al., 2017; Di Tano et al., 2020; Hou et al., 2020). In this section, vitamins B, C, E, and K are essential for regulating cellular homeostasis that directly linked to ferroptotic cell death has been overviewed.

### Vitamin B

**FIGURE 2:**
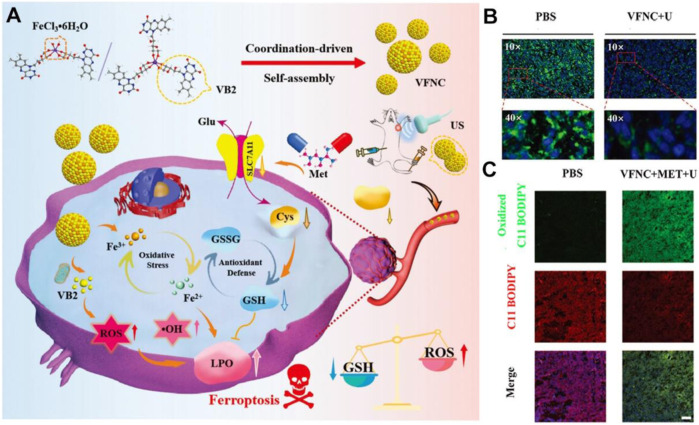
Schematic illustration of VFNC-based ferroptosis therapy towards triple-negative breast cancer. **(A)** Schematic of the preparation process and therapeutic mechanism of the VFNC nanoplatform. **(B)** Immunofluorescence staining of GPX4. **(C)** CLSM images of LPO accumulation. Adapted with permission from Zhou M. et al. (2023). Copyright (2023) John Wiley & Sons, Inc.

The vitamin B family generally serves as enzyme cofactors carrying out essential cellular functions, such as energy metabolism, DNA synthesis, and phospholipids metabolism (Lindschinger et al., 2019). B-group vitamins are water-soluble; the metabolic half-life is only a few hours and must be supplemented daily in most cases (Tardy et al., 2020). Existing evidence indicates that insufficient or excessive supply of B vitamins may elicit oxidative stress and modulate the expression of numerous pathogenic genes (Kennedy, 2016; Peterson et al., 2020). As a proof-of-concept, assembled Vitamin B2 nanocrystals have been exploited to favor the massive production of ROS under blue light irradiation taking advantage of optical waveguiding properties (Fei et al., 2019). Inspired by these interesting properties, Zhou et al. subtly reported the strategy by combining Vitamin B2 and trivalent iron in a self-assembly nanostructure (VFNC) for multiple ferroptosis regulation (Figure 2A; Zhou M. et al., 2023). This vitamin B2-ferric nanocomplex exhibited appropriate size and favorable biocompatibility. Activated by ultrasound, VB2 could contribute to producing a significant quantity of lethal ^1^O_2_ to inhibit cell proliferation. Simultaneously, the disassembled nanocomplex will release Fe^3+^ ion, which could generate •OH through the Fenton reaction and destroy the System Xc^−^-GSH-GPX4 antioxidant defense system. The downregulated GPX4 and SLC7A11 protein and more LPO accumulation suggested the effective facilitation of metformin to VFNC-based ferroptosis therapy (Figures 2B,C). The synergistic sonodynamic/ferroptotic therapy and the coactivation effect of metformin could exert a superadditive anti-tumor effect by completely disintegrating the redox homeostasis and antioxidant defense system of triple-negative breast cancer. Overall, this coordination-driven self-assembly system provides a general strategy for nutritional vitamins in clinical applications.

**FIGURE 3:**
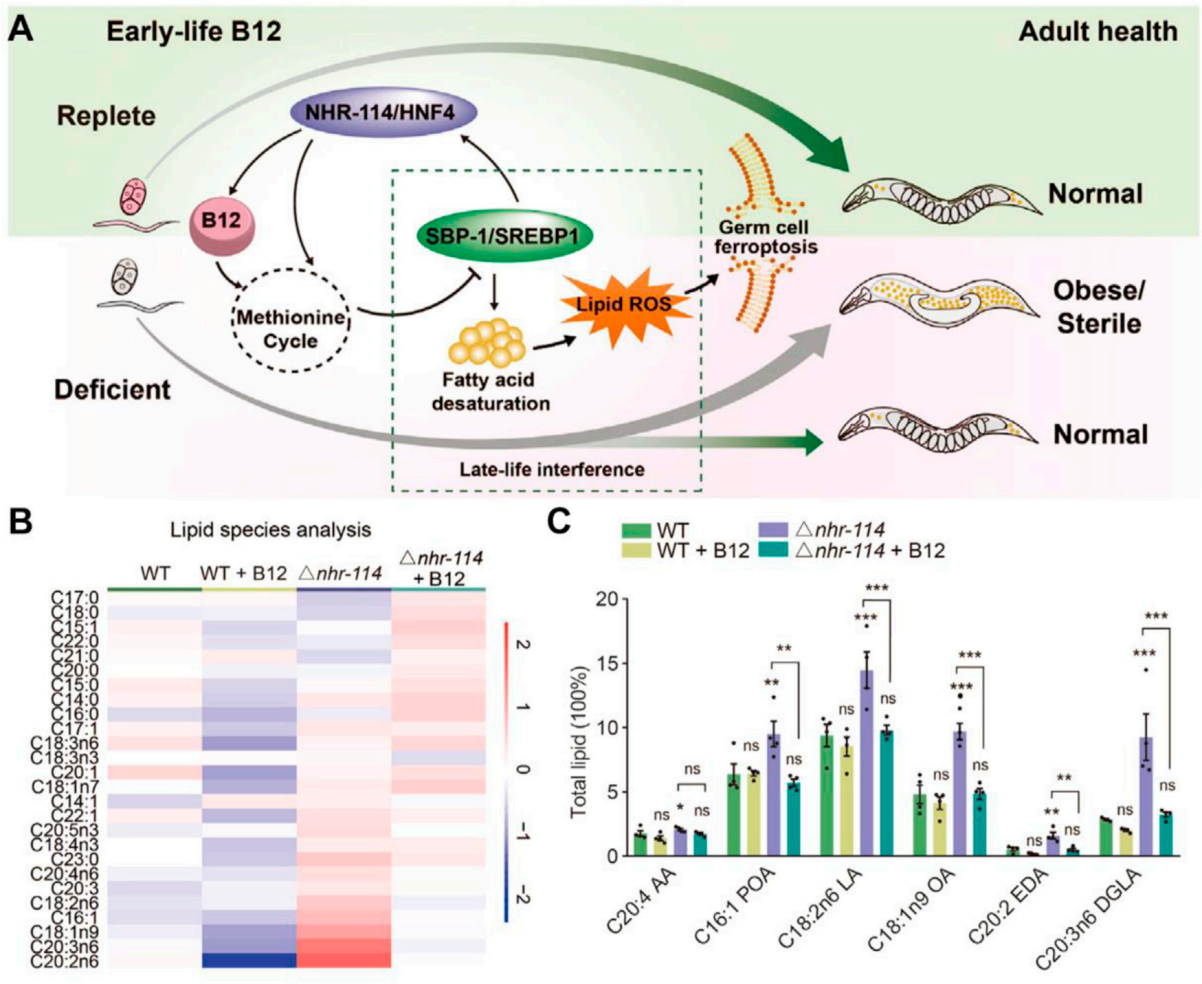
**(A)** Mechanism of action of early-life B12 in programming of adult health. **(B,C)** Heatmap and quantification of lipid species with maternal B12 supplementation. Reproduced with permission (Qin et al., 2022). Copyright 2022, Cell Press.

Recently, Qin et al. established the impact of early vitamin B12 insufficiency in ferroptosis induction (Figure 3A; Qin et al., 2022). Vitamin B12 sufficiency has no adverse impact on germ cells; however, early vitamin B12 deficiency leads to ferroptosis in germ cells and causes obesity and infertility. Mechanistically, vitamin B12 deficiency directly affects the methionine cycle-SBP-1/sterol regulatory element-binding protein-1 (SREBP1)-adipogenesis axis, resulting in the accumulation of polyunsaturated fatty acids and heightened peroxidation levels, which was significantly suppressed by the addition of B12 supplementation (Figures 3B,C). Overall, this study highlights the relationship between vitamin B12 deficiency and ferroptosis in early life, providing a potential target for the late-life treatment of the disease in adulthood.

### Vitamin C

In biological systems, vitamin C exists in different redox forms, including ascorbate, ascorbate radical, dehydroascorbic acid (DHA), 2,3-l-diketoglutonate (2,3-DKG), oxalic acid, and so on. Specifically, the ascorbyl radical is generated upon donating electrons to ferric iron, leading to the formation of superoxide anion (•O_2_^−^) and H_2_O_2_, while ascorbate in pharmacologic concentration can contribute to the suspended formation of •OH (Szarka et al., 2021).

**FIGURE 4:**
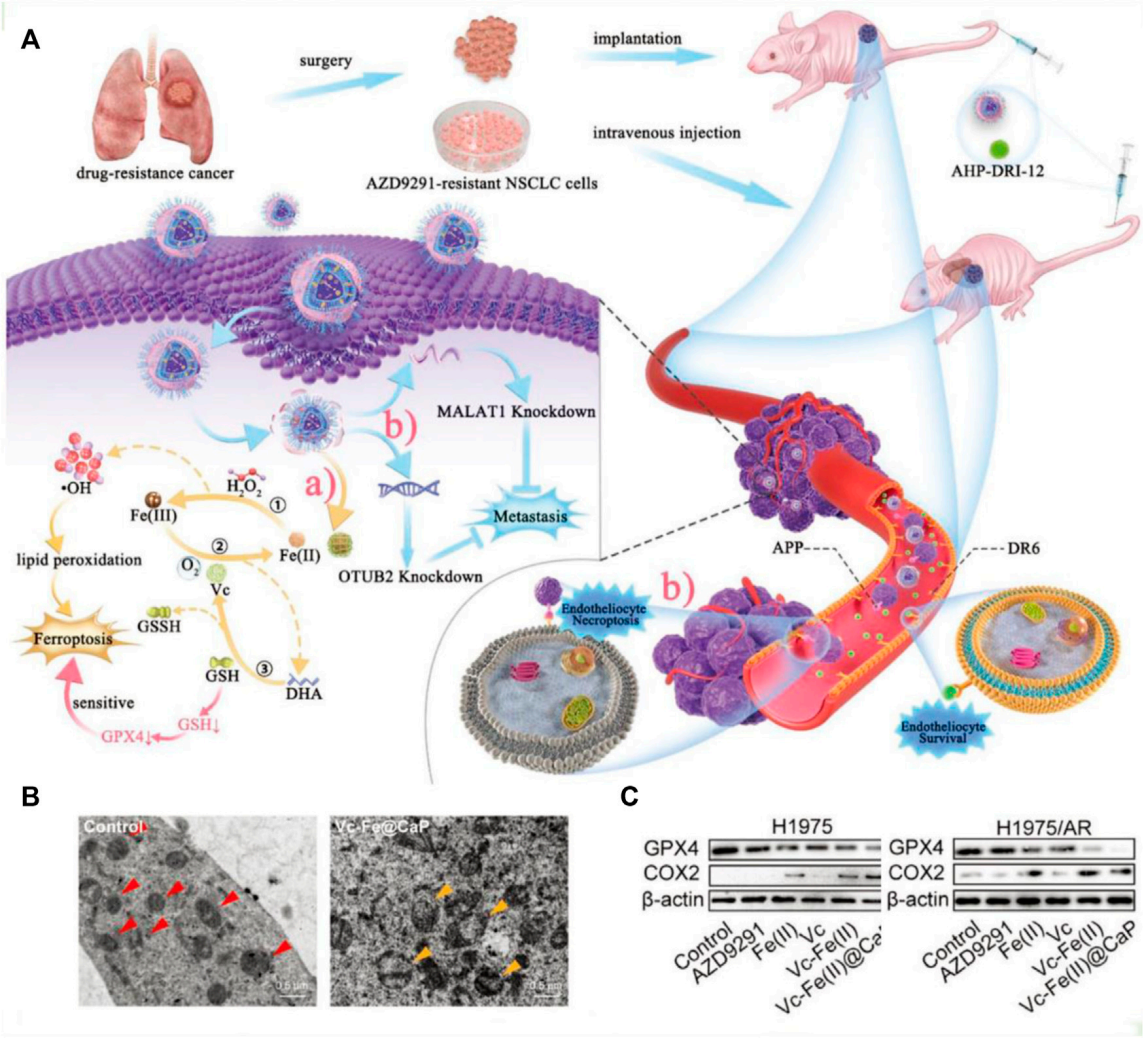
**(A)** Schematic illustration of the tumoricidal effect of combination therapy of VF/S/A@CaP and AHP-DRI-12. **(B)** The cryo-electron microscope images of H1975/AR cells from the control group or Vc–Fe(II)@CaP group. The red triangle serves as a marker for healthy mitochondria, whereas the yellow triangle signifies vacuolated mitochondria that have sustained severe oxidative damage. **(C)** The expression levels of GPX4 and COX2 in both H1975 and H1975/AR cells have been assayed. Reproduced with permission (Wang et al., 2022). Copyright (2022), John Wiley & Sons, Inc.

In addition, vitamin C can affect iron metabolism by inhibiting the degradation of ferritin, increasing the synthesis of ferritin, and thus promoting iron absorption (Wang X. et al., 2021). This cellular death pathway shows similar characteristics to erastin-mediated ferroptosis (Liu et al., 2022). Although vitamin C as an anticancer therapeutic is still up for debate, ongoing recent clinical trials are investigating the combination of pharmacological ascorbate with concurrent radiochemotherapy. Various studies have demonstrated the enhanced therapeutic effect of employing vitamin C to treat troublesome non-small cell lung cancer (NSCLC) (Ou et al., 2020). Recently, a nanocatalytic sensitizer strategy with vitamin C involved has been proposed for treating metastatic NSCLC (Figure 4A; Wang et al., 2022). In this nanotherapy platform, the rational encapsulation of vitamin C and ferrous iron within the liposome enabled the reversible redox cycle. The ferrous ions can interact with hydrogen peroxide, yielding highly reactive •OH radicals, inducing lipid peroxidation (LPO) and ultimately triggering ferroptosis. As shown in Figure 4B, cells underwent oxidative stress and formed vacuolated mitochondria after drug administration. In addition, vitamin C is oxidized to produce DHA, which consumes intracellular GSH, resulting in decreased GPX4 expression (Figure 4C). Moreover, this effective nanocatalytic platform can simultaneously solve the problem of resistance to the kinase inhibitor oxitinib in metastasis NSCLC.

**FIGURE 5:**
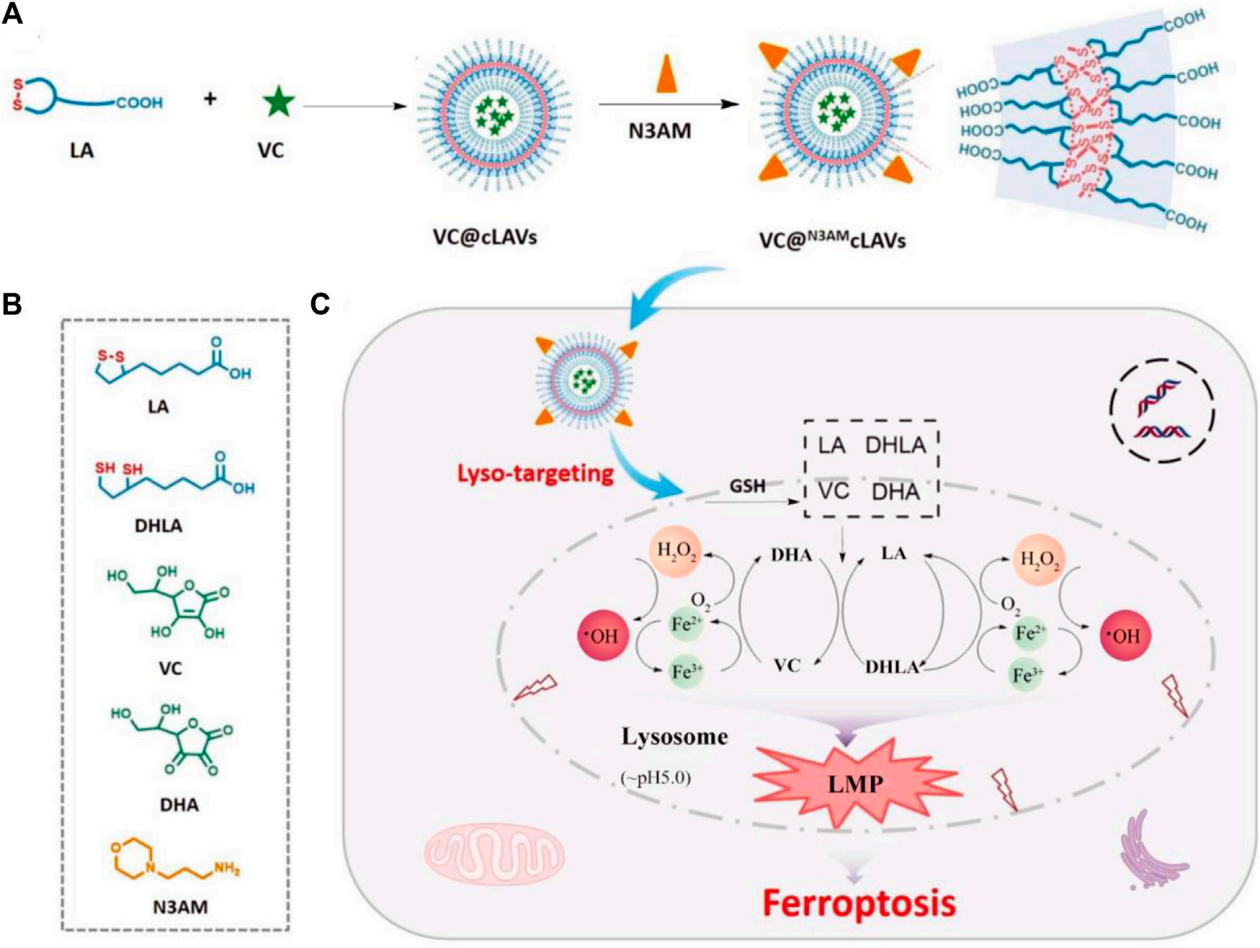
The schematic illustration outlines the process of enhancing ROS-mediated lysosomal membrane permeabilization for cancer ferroptosis therapy. **(A)** The design methods of VC@^N3AM^cLAVs. **(B)** The various molecular structures from the composition of VC@^N3AM^cLAVs. **(C)** Lysosome-targeting process of VC@^N3AM^cLAVs and the specific mechanism to promote ROS-mediated lysosomal membrane permeabilization for efficient ferroptosis therapy. Adapted with permission from Chen et al. (2022). Copyright (2023) John Wiley & Sons, Inc.

It is a widely recognized fact that mitochondria serve as the primary organelles responsible for iron metabolism within cells. Consequently, the development of mitochondria-targeting ferroptosis platforms has demonstrated promising antitumor effects (Gan et al., 2023). Nevertheless, recent studies have revealed that the rational design of lysosome-targeting systems capable of inducing ferroptosis may yield significant outcomes. These findings broaden our understanding of the complex mechanisms involved in ferroptosis and suggest novel therapeutic strategies for targeting this process. For instance, Chen et al. successfully synthesized a lysosome-targeting ferroptosis nanoplatform (Figure 5; Chen et al., 2022). The synthesis process and chemical structures of the components of VC@N3AMcLAVs are thoroughly outlined in Figures 5A,B. Notably, the incorporation of morpholine enabled the nanotherapeutic platform to specifically target lysosomes. Once inside the lysosome, the nanosystem interacts with excess GSH leading to the reduction of lipoic acid and oxidation of vitamin C. Additionally, the oxygen present in the lysosome is converted to hydrogen peroxide. Furthermore, this efficient redox cycle facilitates the conversion of ferric ions to ferrous ions through the degradation of ferritin within the lysosome, further enhancing the antitumor effects of ferroptosis. The produced excess hydrogen peroxide will interact with the ferrous ions to produce highly oxidizing •OH. Due to the depletion of GSH and deficiency of catalase, large numbers of ROS will destroy the redox homeostasis of lysosomes and result in the complete breakdown of the lysosomes membrane, inducing the release of proteolytic enzymes and other substances into the cytosol. This lysosomal membrane permeabilization will cause irreversible damage to tumor cells, resulting in efficient ferroptosis therapy (Figure 5C). The system successfully uses the interaction between the external vitamin C and the intracellular GSH to cause the irreversible death of tumor cells, which provides a new option for effective anticancer therapy. This design offers a promising approach for precision delivery and enhanced antitumor effects through ferroptosis induction in lysosomes.

Recently, vitamin C at pharmaceutical dosage has been demonstrated to be efficient in strengthening the antineoplastic effect by eliciting host immune systems (Ngo et al., 2019). In particular, high-dose vitamin C treatment exhibits great synergy in potentiating the efficacy of immune checkpoint therapy (Magrì et al., 2020). Exploring vitamin C-mediated immunomodulation, Yu et al. proposed hybrid core-shell vesicles (HCSVs) to augment ferroptosis-based therapy by an exogenous circularly polarized magnetic field, as well as responsive magnetic resonance imaging capability (Yu et al., 2020). The HCSVs were constructed by encapsulating ascorbic acid within poly lactic-co-glycolic acid, and the iron oxide nanocubes were embedded into the outer layer. Under the external magnetic field, the vesicle structure is destroyed along with the release of ascorbic acid to interact with magnetic iron oxide. Ascorbic acid can oxidize ferric ions to produce ferrous ions, which could induce subsequent Fenton chemical reaction processes. Subsequently, the Fenton reaction leads to ferroptosis-mediated cancer inhibition by excessive LPO accumulation. Concurrently, the elevated oxidative stress could induce the activation of dendritic cells and infiltration of cytotoxic T lymphocytes to exert an immunomodulatory effect. During the reduction of magnetic iron oxide by ascorbic acid, the iron-based magnetic resonance imaging signal provides an efficient strategy for visualization of the Fenton reaction.

### Vitamin E

As an endogenous radical-trapping antioxidant, vitamin E is a general term for tocopherol substances exhibiting multiple health benefits. Vitamin E comprises different forms of tocopherol and tocotrienol generally functions by the electron-transport coupled enzymatic mechanisms to prevent oxidative damage (Peh et al., 2016; Blaner et al., 2021). For ferroptosis execution, vitamin E actively binds to the PUFA substrate to favor the protective effects against LPO propagation (Hu et al., 2021). Recently, Zhang et al. revealed the anti-ferroptotic defense of nutrient vitamin E for chronic neurological diseases (Zhang X. et al., 2022). Mechanistically, vitamin E exerts neuroprotective effects largely by inhibiting LOX-15, and vitamin E pretreatment can reduce neuronal damage in pentylenetetrazol-induced epileptic rats. In addition, tetrahydrobiopterin (BH4) has been screened out to protect lipids from autoxidation in synergy with vitamin E (Soula et al., 2020). Metabolism-focused screens demonstrated that BH4 biosynthesis is essential for GPX4 inhibition, and can regenerate from its oxidative forms upon dihydrofolate reductase (DHFR), maintaining sustained anti-ferroptotic function. According to the autoxidations of STY-BODIPY, BH4 had similar ferroptosis-inhibitory effects in comparison to α-tocopherol (α-TOH). In the liposomal system, the combination of BH4 and α-TOH yielded superior anti-ferroptosis defenses, partially because of the scavenging of α-TOH-derived radicals.

### Vitamin K

**FIGURE 6:**
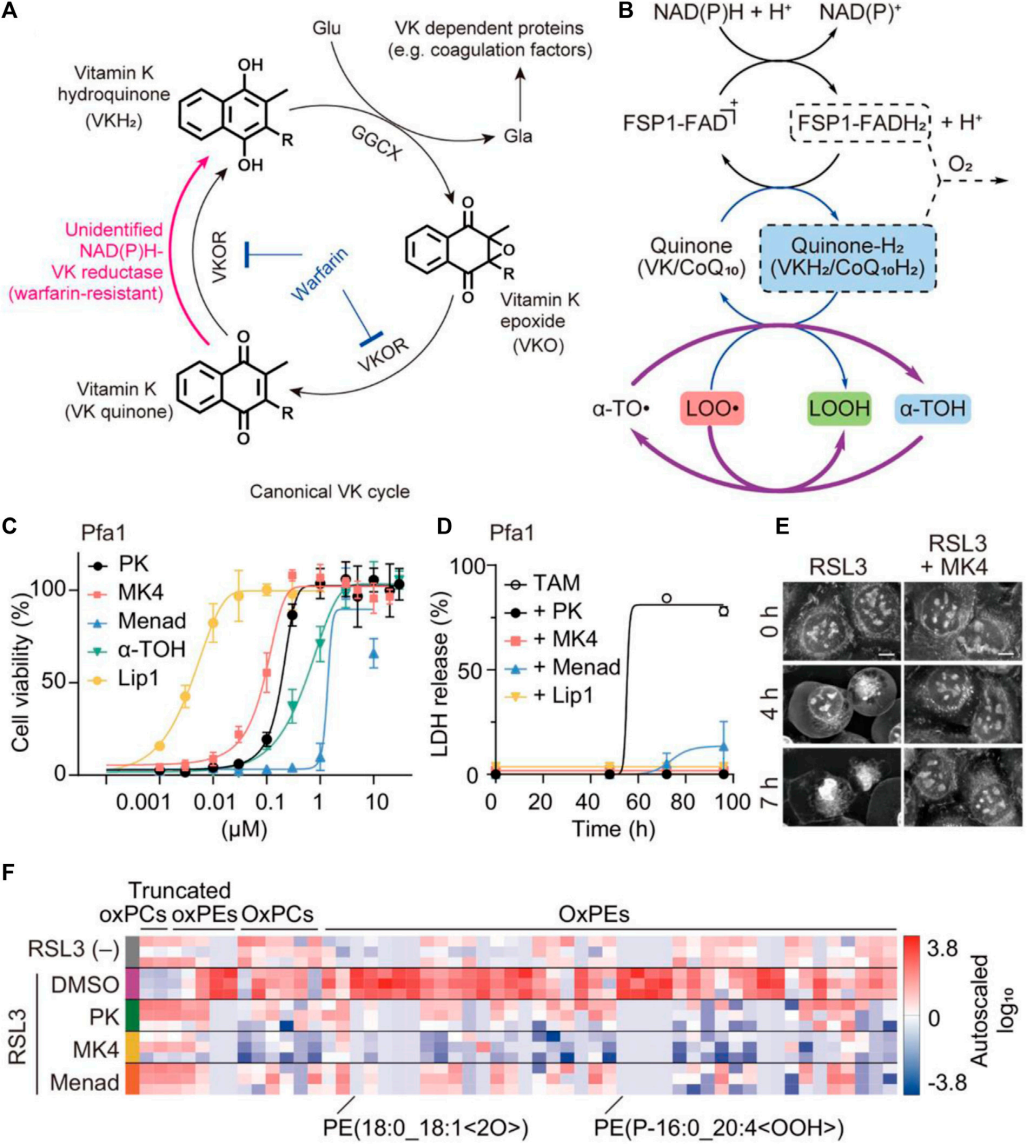
The anti-ferroptotic mechanism of non-canonical vitamin K cycle. **(A)** Mechanism of vitamin K supplying VKH2 to GGCX via the alternative vitamin K reduction pathway. **(B)** The possible recycling pathway of α-tocopherol via the CoQ10/VK-hydroquinone transformation process. **(C)** The cell viability and **(D)** lactate dehydrogenase (LDH) release from GPX4-KO mouse embryonic fibroblasts (Pfa1 cells) treated with different conditions (three forms of vitamin K and liproxstatin-1 (Lip1) as a ferroptosis inhibitor). **(E)** The cellular morphology images of RSL3-treated HT-1080 cells were observed irrespective of whether MK4 was present. **(F)** Proportional levels of oxidized phospholipids are visualized through heatmaps during an 8-h exposure to RSL3 in Pfa1 cells (Mishima et al., 2022). Copyright (2021) Springer Nature.

Vitamin K is a general term referring to a group of structurally related compounds that possess a common naphthoquinone ring. These compounds encompass both natural and synthetic forms, including phylloquinone (known as vitamin K1) found primarily in plants, menaquinones (vitamin K2) found in animal products, and synthetic analogs such as vitamin K3 and menaquinone-4 (MK4) (Welsh et al., 2022). The primary biological role of vitamin K is serving as a cofactor for the γ-carboxylation process, which is crucial in preventing excessive hemorrhaging and osteoporosis. This cofactor function is vital for maintaining bone health and blood coagulation (Wallin and Hutson, 2004; Shearer and Okano, 2018). Recently, vitamin K1 as the predominant dietary form has been investigated as a potential ferroptosis suppressor (Kolbrink et al., 2022). In 2022, Kolbrink et al. proved that vitamin K1 can participate in ferroptosis inhibitory systems, providing pharmacological control of ischemia-reperfusion injury (Kolbrink et al., 2022). In the NIH3T3 cells, the frequency of cell death in the RSL3 group and RSL3 + vitamin K1 group was observed to be 80.8% and 2.2%, respectively. Both vitamin K1 (10 μM) and ferrostatin-1 (1 μM) could remarkably counteract the cytotoxicity by inhibiting RSL3-induced ferroptosis at the cellular level. As evidenced by the phosphatidylserine accessibility, vitamin K1 can also reverse ferroptotic cell death by inhibition of FSP1 and mitochondrially dihydroorotate dehydrogenase (DHODH). For renal ischemia-reperfusion, vitamin K1 had a protective effect on acute kidney injury by inhibiting tubular necrosis and ACSL4 expression. Overall, vitamin K1 demonstrated great therapeutic potential for many diseases associated with ferroptosis by targeting multiple inhibitory pathways. Apart from vitamin K1, variant synthetic menadione has recently been proven to rescue cells from ferroptosis by GPX4 deletion (Mishima et al., 2022). As depicted in Figure 6A, vitamin K elicits ferroptotic inhibitory function that largely depends on FSP1-mediated reduction. In the canonical vitamin K cycle, vitamin K is typically reduced to vitamin K hydroquinone (VKH_2_), which is generally recognized as the radical-trapping antioxidant (Figure 6A). However, VKH_2_ is easily oxidized to vitamin K epoxide and then converted to vitamin K quinone losing antioxidant activity, this process is also known as warfarin poisoning. Intriguingly, FSP1 was able to form a direct cycle from vitamin K to VKH_2_, maintaining lipid radical-trapping activity to attenuate ferroptosis (Figure 6B). In pfa1 cells, MK4 is the most efficacious form of vitamin K derivatives in preventing ferroptosis induced by GPX4 deletion or knockdown (Figure 6C). The significantly reduced release percentage of lactate dehydrogenase further indicated the protective effect of three forms of vitamin K derivative (PK/MK4/Menad) against cell death (Figure 6D). In particular, the presence of MK4 was able to successfully prevent HT-1080 cells from undergoing pathologic changes associated with ferroptosis induced by RSL3 (Figure 6E). Lipid peroxidation is a key process in ferroptosis, where the accumulation of lipid peroxides leads to membrane damage and ultimately cell death. By reducing the level of lipid peroxide, the three forms of vitamin K can mitigate the progression of ferroptosis (Figure 6F). This finding suggests that vitamin K may act as an efficient antioxidant or regulate cellular processes that lead to lipid peroxidation, thereby protecting cells from ferroptosis. Overall, vitamin K metabolism is thought to play a critical role in ferroptosis-associated disease, acting as a natural active ingredient in anti-ferroptotic therapy through the non-classical vitamin K cycle.

**FIGURE 7:**
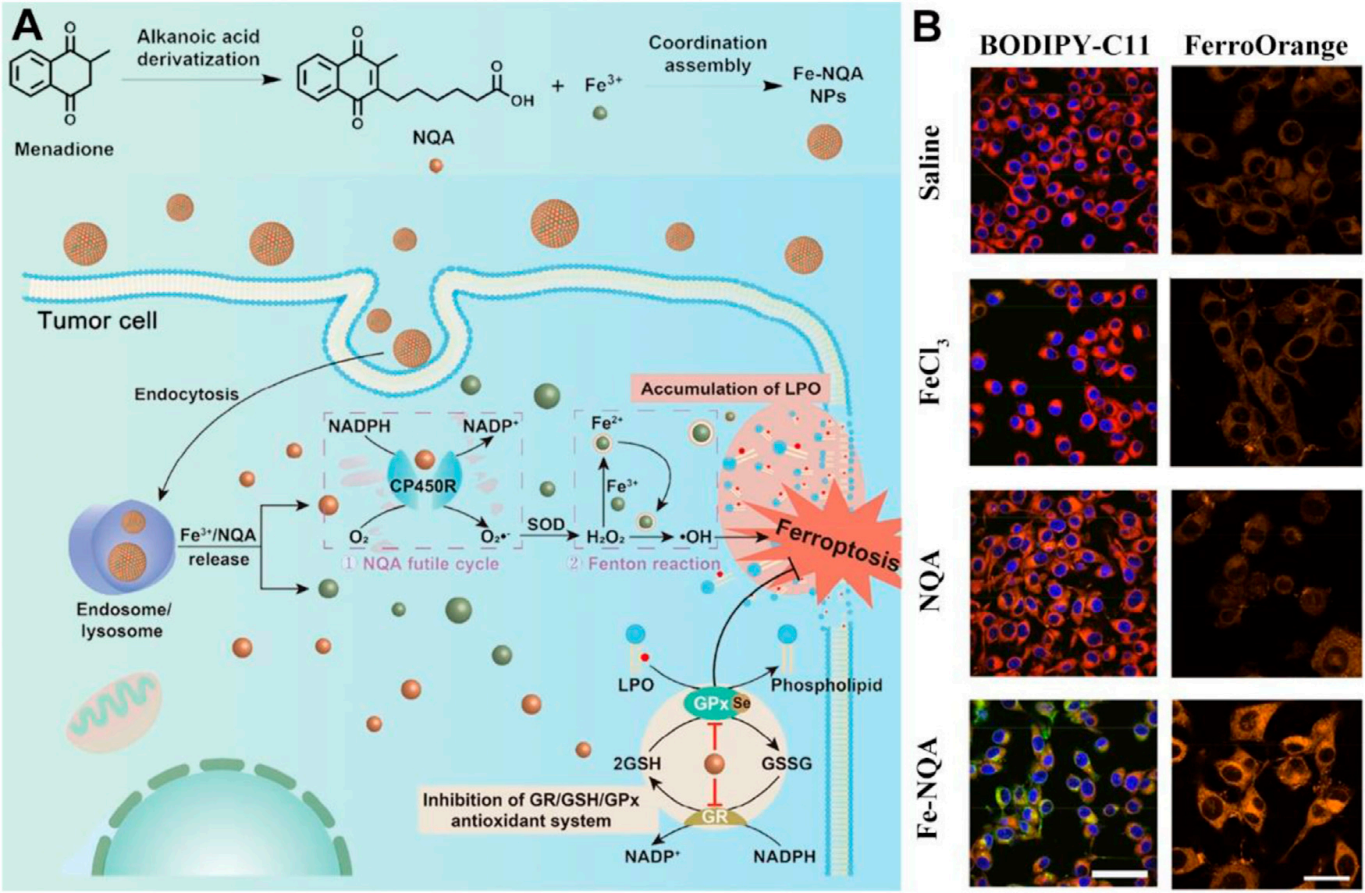
The preparation and action mechanism of Fe-NQA for augmenting tumor ferroptotic therapy. **(A)** Schematic illustration of Fe-NQA NPs induced ferroptotic therapy. **(B)** The LSCM images of LPO and Fe^2+^ detected in CT26 cells after incubation of cells with saline, FeCl_3_, NQA, and Fe-NQA NPs (scale bar: 50 μm). Adapted with permission from Zhang Z. et al. (2020). Copyright (2020) Springer Nature.

Furthermore, the synthetic variant menadione (vitamin K3) is known to be a notable anti-inflammatory drug, which can exacerbate ROS generation to achieve therapeutic effects against bacteria or viruses at appropriate wavelengths (Wang R. et al., 2021). Given that, vitamin K3 and its derivatives have been employed in the field of ROS-based nanomedicine aiming at achieving improved therapeutic index (Yang et al., 2018; Wellington et al., 2020; Chauhan et al., 2023). Utilizing iron ion therapy, nanoscale coordination polymers known as Fe-NQA have been suggested as a potential tumor treatment method, capable of initiating the Fenton reaction while simultaneously suppressing antioxidant activity (Figure 7; Zhang Z. et al., 2020). The Fe-NQA was prepared by one-step coordination self-assembly of Fe^3+^ and 6-[2-(3-methyl)naphthoquinolyl]hexanoic acid (NQA). The NQA can not only facilitate semiquinone radical formation but also inhibit GPX4 activation by binding cysteine or selenocysteine residues. After internalization, Fe-NQA disassembles in a pH-responsive manner to form a sustained cycle favoring the Fenton reaction by catalyzed cytochrome P450 reductase. Additionally, Fe-NQA sustains GPX4 inactivation, and this cellular death can be reversed by ferroptosis inhibitors. The staining process employed FerroOrange and BODIPY-C11 probes, revealing that the level of lipid peroxide (LPO) in CT26 cells incubated with Fe-NQA nanoparticles was notably elevated compared to the group treated with normal saline (Figure 7B). Taken together, Fe-NQA can synergistically initiate ferroptosis execution, enabling efficient tumor regression, metastasis prevention, and radiotherapy sensitization.

## Conclusion and perspectives

Ferroptosis has achieved unprecedented outcomes since coined in 2012, and therapeutic intervention has been demonstrated to be tightly linked to neurodegeneration, viral infection, and cardiovascular disease. Especially for malignant tumors, proferroptotic stimulation has sufficiently indicated significant impacts on the inhibition, development, and metastasis of tumor cells. Existing research has sufficiently indicated ferroptosis inducers or inhibitors, and compartmentalized by regulatory networks involves iron, amino acids, and lipids metabolism. Elucidating these metabolism networks may be critical for therapeutic benefit in various ferroptosis-associated pathophysiological contexts. However, the complicated ferroptosis-based crosstalk between multiple nutrients and metabolic regulation of cellular homeostasis is still limited, especially in the field of vitamin-based essential nutrients.

The administration of nutritional vitamin supplements could potentially serve as an effective approach to modulate sublethal ferroptosis-associated disorders. To date, essential vitamins enabling the regulation of redox homeostasis and LPO generally depend on perturbing ROS hemostasis. However, the current understanding of ROS biology is orchestrated by complex molecular pathways comprising different cell death modalities. Conversely, the interplay between the mechanism of ferroptotic cell death and its associated factors with nutrient signaling remains relatively unexplored.

Furthermore, metabolism regulation of iron availability, cystine deprivation, and ROS biology has yielded insight into the nutrient vitamins in remodeled biological hemostasis. However, interconnected metabolism pathways are highly complex, which is also referred to as metabolic plasticity. The modulation of ferroptosis may involve various endogenous substrates, and vitamins could potentially play a role in other forms of controlled cell death. Nevertheless, the intricate interactions and transitions between ferroptosis and other cell death mechanisms remain elusive.

Finally, the pathophysiological signals of ferroptotic cell death must be unambiguously elucidated stem from the fact that these effectors are directly related to the therapeutic potential of ferroptosis-associated disease. Stratifying ferroptosis sensitivity in patients holds promise for conferring significant health benefits in clinical practice. Current oxidative lipidomics is largely time-consuming, while other reliable molecular imaging methodologies are relatively not enough to favor real-time monitoring of this regulated cell death modality. There is an urgent need for advanced techniques and reagents capable of monitoring treatment processes, in order to rigorously assess the pathological significance of ferroptosis.

## References

[B1] Andrews N. C. Schmidt P. J. (2007). Iron homeostasis. Annu. Rev. Physiol. 69, 69–85. 10.1146/annurev.physiol.69.031905.164337 17014365

[B2] Bao W. D. Pang P. Zhou X. T. Hu F. Xiong W. Chen K. (2021). Loss of ferroportin induces memory impairment by promoting ferroptosis in Alzheimer's disease. Cell Death Differ. 28, 1548–1562. 10.1038/s41418-020-00685-9 33398092 PMC8166828

[B3] Bao W. D. Zhou X. T. Zhou L. T. Wang F. Yin X. Lu Y. (2020). Targeting miR-124/Ferroportin signaling ameliorated neuronal cell death through inhibiting apoptosis and ferroptosis in aged intracerebral hemorrhage murine model. Aging Cell 19, e13235. 10.1111/acel.13235 33068460 PMC7681046

[B4] Bazinet R. P. Layé S. (2014). Polyunsaturated fatty acids and their metabolites in brain function and disease. Nat. Rev. Neurosci. 15, 771–785. 10.1038/nrn3820 25387473

[B5] Blaner W. S. Shmarakov I. O. Traber M. G. (2021). Vitamin A and vitamin E: will the real antioxidant please stand up? Annu. Rev. Nutr. 41, 105–131. 10.1146/annurev-nutr-082018-124228 34115520

[B6] Bogdan A. R. Miyazawa M. Hashimoto K. Tsuji Y. (2016). Regulators of iron homeostasis: new players in metabolism, cell death, and disease. Trends biochem. Sci. 41, 274–286. 10.1016/j.tibs.2015.11.012 26725301 PMC4783254

[B7] Chauhan A. Anjaly K. Saini A. Kumar R. Kuanr B. K. Sharma D. (2023). Vitamin k3-loaded magnetic nanoparticle-mediated synergistic magnetothermodynamic therapy evokes massive ROS and immune modulation for augmented antitumor potential. ACS Appl. Mater Interfaces 15, 27515–27532. 10.1021/acsami.3c01702 37264797

[B8] Chen Y. Yang Z. Wang S. Ma Q. Li L. Wu X. (2022). Boosting ROS‐mediated lysosomal membrane permeabilization for cancer ferroptosis therapy. Adv. Healthc. Mater. 12, e2202150. 10.1002/adhm.202202150 36408929

[B9] Di Tano M. Raucci F. Vernieri C. Caffa I. Buono R. Fanti M. (2020). Synergistic effect of fasting-mimicking diet and vitamin C against KRAS mutated cancers. Nat. Commun. 11, 2332. 10.1038/s41467-020-16243-3 32393788 PMC7214421

[B10] Dixon S. j. Lemberg K. m. Lamprecht M. r. Skouta R. Zaitsev E. m. Gleason C. e. (2012). Ferroptosis: an iron-dependent form of nonapoptotic cell death. Cell 149, 1060–1072. 10.1016/j.cell.2012.03.042 22632970 PMC3367386

[B11] Dyall S. C. Balas L. Bazan N. G. Brenna J. T. Chiang N. Da Costa Souza F. (2022). Polyunsaturated fatty acids and fatty acid-derived lipid mediators: recent advances in the understanding of their biosynthesis, structures, and functions. Prog. Lipid Res. 86, 101165. 10.1016/j.plipres.2022.101165 35508275 PMC9346631

[B12] Fang Y. Chen X. Tan Q. Zhou H. Xu J. Gu Q. (2021). Inhibiting ferroptosis through disrupting the NCOA4–FTH1 interaction: a new mechanism of action. ACS Cent. Sci. 7, 980–989. 10.1021/acscentsci.0c01592 34235259 PMC8227600

[B13] Fei J. Dai L. Gao F. Zhao J. Li J. (2019). Assembled vitamin B2 nanocrystals with optical waveguiding and photosensitizing properties for potential biomedical application. Angew. Chem. Int. Ed. Engl. 58, 7254–7258. 10.1002/anie.201900124 30912208

[B14] Gan B. (2022). ACSL4, PUFA, and ferroptosis: new arsenal in anti-tumor immunity. Signal Transduct. Target Ther. 7, 128. 10.1038/s41392-022-01004-z 35459217 PMC9033814

[B15] Gan H. Huang X. Luo X. Li J. Mo B. Cheng L. (2023). A mitochondria-targeted ferroptosis inducer activated by glutathione-responsive imaging and depletion for triple negative breast cancer theranostics. Adv. Healthc. Mater 12, e2300220. 10.1002/adhm.202300220 37204240

[B16] Gong Y. Fan Z. Luo G. Yang C. Huang Q. Fan K. (2019). The role of necroptosis in cancer biology and therapy. Mol. Cancer 18, 100. 10.1186/s12943-019-1029-8 31122251 PMC6532150

[B17] Hashidate-Yoshida T. Harayama T. Hishikawa D. Morimoto R. Hamano F. Tokuoka S. M. (2015). Fatty acid remodeling by LPCAT3 enriches arachidonate in phospholipid membranes and regulates triglyceride transport. Elife 4, e06328. 10.7554/eLife.06328 25898003 PMC4436788

[B18] Hassannia B. Vandenabeele P. Vanden Berghe T. (2019). Targeting ferroptosis to iron out cancer. Cancer Cell 35, 830–849. 10.1016/j.ccell.2019.04.002 31105042

[B19] Hou X. Zhang X. Zhao W. Zeng C. Deng B. Mccomb D. W. (2020). Vitamin lipid nanoparticles enable adoptive macrophage transfer for the treatment of multidrug-resistant bacterial sepsis. Nat. Nanotechnol. 15, 41–46. 10.1038/s41565-019-0600-1 31907443 PMC7181370

[B20] Hu Q. Zhang Y. Lou H. Ou Z. Liu J. Duan W. (2021). GPX4 and vitamin E cooperatively protect hematopoietic stem and progenitor cells from lipid peroxidation and ferroptosis. Cell Death Dis. 12, 706. 10.1038/s41419-021-04008-9 34267193 PMC8282880

[B21] Jiang X. Stockwell B. R. Conrad M. (2021). Ferroptosis: mechanisms, biology and role in disease. Nat. Rev. Mol. Cell Biol. 22, 266–282. 10.1038/s41580-020-00324-8 33495651 PMC8142022

[B22] Kagan V. E. Mao G. Qu F. Angeli J. P. F. Doll S. Croix C. S. (2016). Oxidized arachidonic and adrenic PEs navigate cells to ferroptosis. Nat. Chem. Biol. 13, 81–90. 10.1038/nchembio.2238 27842066 PMC5506843

[B23] Kennedy D. (2016). B vitamins and the brain: mechanisms, dose and efficacy-A review. Nutrients 8, 68. 10.3390/nu8020068 26828517 PMC4772032

[B24] Kerr J. F. R. Wyllie A. H. Currie A. R. (1972). Apoptosis: a basic biological phenomenon with wideranging implications in tissue kinetics. Br. J. Cancer 26, 239–257. 10.1038/bjc.1972.33 4561027 PMC2008650

[B25] Kolbrink B. Von Samson-Himmelstjerna F. A. Messtorff M. L. Riebeling T. Nische R. Schmitz J. (2022). Vitamin K1 inhibits ferroptosis and counteracts a detrimental effect of phenprocoumon in experimental acute kidney injury. Cell Mol. Life Sci. 79, 387. 10.1007/s00018-022-04416-w 35763128 PMC9239973

[B26] Koppula P. Zhang Y. Zhuang L. Gan B. (2018). Amino acid transporter SLC7A11/xCT at the crossroads of regulating redox homeostasis and nutrient dependency of cancer. Cancer Commun. 38, 12. 10.1186/s40880-018-0288-x PMC5993148 29764521

[B27] Koppula P. Zhuang L. Gan B. (2021). Cystine transporter SLC7A11/xCT in cancer: ferroptosis, nutrient dependency, and cancer therapy. Protein & Cell 12, 599–620. 10.1007/s13238-020-00789-5 33000412 PMC8310547

[B28] Lee J. Roh J.-L. (2022). SLC7A11 as a gateway of metabolic perturbation and ferroptosis vulnerability in cancer. Antioxidants 11, 2444. 10.3390/antiox11122444 36552652 PMC9774303

[B29] Lei G. Zhuang L. Gan B. (2022). Targeting ferroptosis as a vulnerability in cancer. Nat. Rev. Cancer 22, 381–396. 10.1038/s41568-022-00459-0 35338310 PMC10243716

[B30] Liang C. Zhang X. Yang M. Dong X. (2019). Recent progress in ferroptosis inducers for cancer therapy. Adv. Mater 31, e1904197. 10.1002/adma.201904197 31595562

[B31] Liang D. Minikes A. M. Jiang X. (2022). Ferroptosis at the intersection of lipid metabolism and cellular signaling. Mol. Cell 82, 2215–2227. 10.1016/j.molcel.2022.03.022 35390277 PMC9233073

[B32] Lin L. Wang S. Deng H. Yang W. Rao L. Tian R. (2020). Endogenous labile iron pool-mediated free radical generation for cancer chemodynamic therapy. J. Am. Chem. Soc. 142, 15320–15330. 10.1021/jacs.0c05604 32820914

[B33] Lindschinger M. Tatzber F. Schimetta W. Schmid I. Lindschinger B. Cvirn G. (2019). A randomized pilot trial to evaluate the bioavailability of natural versus synthetic vitamin B complexes in healthy humans and their effects on homocysteine, oxidative stress, and antioxidant levels. Oxid. Med. Cell Longev. 2019, 6082613. 10.1155/2019/6082613 31915511 PMC6930747

[B34] Liu Y. Huang P. Li Z. Xu C. Wang H. Jia B. (2022). Vitamin C sensitizes pancreatic cancer cells to erastin-induced ferroptosis by activating the AMPK/Nrf2/HMOX1 pathway. Oxid. Med. Cell Longev. 2022, 5361241. 10.1155/2022/5361241 35915609 PMC9338737

[B35] Magrì A. Germano G. Lorenzato A. Lamba S. Chilà R. Montone M. (2020). High-dose vitamin C enhances cancer immunotherapy. Sci. Transl. Med. 12, eaay8707. 10.1126/scitranslmed.aay8707 32102933

[B36] Mishima E. Ito J. Wu Z. Nakamura T. Wahida A. Doll S. (2022). A non-canonical vitamin K cycle is a potent ferroptosis suppressor. Nature 608, 778–783. 10.1038/s41586-022-05022-3 35922516 PMC9402432

[B37] Mou Y. Wang J. Wu J. He D. Zhang C. Duan C. (2019). Ferroptosis, a new form of cell death: opportunities and challenges in cancer. J. Hematol. Oncol. 12, 34. 10.1186/s13045-019-0720-y 30925886 PMC6441206

[B38] Ngo B. Van Riper J. M. Cantley L. C. Yun J. (2019). Targeting cancer vulnerabilities with high-dose vitamin C. Nat. Rev. Cancer 19, 271–282. 10.1038/s41568-019-0135-7 30967651 PMC6526932

[B39] Ou J. Zhu X. Chen P. Du Y. Lu Y. Peng X. (2020). A randomized phase II trial of best supportive care with or without hyperthermia and vitamin C for heavily pretreated, advanced, refractory non-small-cell lung cancer. J. Adv. Res. 24, 175–182. 10.1016/j.jare.2020.03.004 32368355 PMC7190757

[B40] Peh H. Y. Tan W. S. Liao W. Wong W. S. (2016). Vitamin E therapy beyond cancer: tocopherol versus tocotrienol. Pharmacol. Ther. 162, 152–169. 10.1016/j.pharmthera.2015.12.003 26706242

[B41] Peng F. Liao M. Qin R. Zhu S. Peng C. Fu L. (2022). Regulated cell death (RCD) in cancer: key pathways and targeted therapies. Signal Transduct. Target. Ther. 7, 286. 10.1038/s41392-022-01110-y 35963853 PMC9376115

[B42] Peterson C. T. Rodionov D. A. Osterman A. L. Peterson S. N. (2020). B vitamins and their role in immune regulation and cancer. Nutrients 12, 3380. 10.3390/nu12113380 33158037 PMC7693142

[B43] Qi Y. Zhang X. Wu Z. Tian M. Chen F. Guan W. (2021). Ferroptosis regulation by nutrient signalling. Nutr. Res. Rev. 35, 282–294. 10.1017/S0954422421000226 34233775

[B44] Qin S. Wang Y. Li L. Liu J. Xiao C. Duan D. (2022). Early-life vitamin B12 orchestrates lipid peroxidation to ensure reproductive success via SBP-1/SREBP1 in *Caenorhabditis elegans* . Cell Rep. 40, 111381. 10.1016/j.celrep.2022.111381 36130518

[B45] Riegman M. Sagie L. Galed C. Levin T. Steinberg N. Dixon S. J. (2020). Ferroptosis occurs through an osmotic mechanism and propagates independently of cell rupture. Nat. Cell Biol. 22, 1042–1048. 10.1038/s41556-020-0565-1 32868903 PMC7644276

[B46] Rizzollo F. More S. Vangheluwe P. Agostinis P. (2021). The lysosome as a master regulator of iron metabolism. Trends Biochem. Sci. 46, 960–975. 10.1016/j.tibs.2021.07.003 34384657

[B47] Saghiri M. A. Asatourian A. Ershadifar S. Moghadam M. M. Sheibani N. (2017). Vitamins and regulation of angiogenesis: [A, B1, B2, B3, B6, B9, B12, C, D, E, K]. J. Funct. Foods 38, 180–196. 10.1016/j.jff.2017.09.005

[B48] Shearer M. J. Okano T. (2018). Key pathways and regulators of vitamin K function and intermediary metabolism. Annu. Rev. Nutr. 38, 127–151. 10.1146/annurev-nutr-082117-051741 29856932

[B49] Sies H. (2021). Oxidative eustress: on constant alert for redox homeostasis. Redox Biol. 41, 101867. 10.1016/j.redox.2021.101867 33657525 PMC7930632

[B50] Snaebjornsson M. T. Janaki-Raman S. Schulze A. (2020). Greasing the wheels of the cancer machine: the role of lipid metabolism in cancer. Cell Metab. 31, 62–76. 10.1016/j.cmet.2019.11.010 31813823

[B51] Soula M. Weber R. A. Zilka O. Alwaseem H. La K. Yen F. (2020). Metabolic determinants of cancer cell sensitivity to canonical ferroptosis inducers. Nat. Chem. Biol. 16, 1351–1360. 10.1038/s41589-020-0613-y 32778843 PMC8299533

[B52] Stockwell B. R. Jiang X. Gu W. (2020). Emerging mechanisms and disease relevance of ferroptosis. Trends Cell Biol. 30, 478–490. 10.1016/j.tcb.2020.02.009 32413317 PMC7230071

[B53] Stoyanovsky D. A. Tyurina Y. Y. Shrivastava I. Bahar I. Tyurin V. A. Protchenko O. (2019). Iron catalysis of lipid peroxidation in ferroptosis: regulated enzymatic or random free radical reaction? Free Radic. Biol. Med. 133, 153–161. 10.1016/j.freeradbiomed.2018.09.008 30217775 PMC6555767

[B54] Szarka A. Kapuy O. Lőrincz T. Bánhegyi G. (2021). Vitamin C and cell death. Antioxid. Redox Signal. 34, 831–844. 10.1089/ars.2019.7897 32586104

[B55] Tang D. Chen X. Kang R. Kroemer G. (2020). Ferroptosis: molecular mechanisms and health implications. Cell Res. 31, 107–125. 10.1038/s41422-020-00441-1 33268902 PMC8026611

[B56] Tang D. Kang R. Berghe T. V. Vandenabeele P. Kroemer G. (2019). The molecular machinery of regulated cell death. Cell Res. 29, 347–364. 10.1038/s41422-019-0164-5 30948788 PMC6796845

[B57] Tardy A. L. Pouteau E. Marquez D. Yilmaz C. Scholey A. (2020). Vitamins and minerals for energy, fatigue and cognition: a narrative review of the biochemical and clinical evidence. Nutrients 12, 228. 10.3390/nu12010228 31963141 PMC7019700

[B58] Tong X. Tang R. Xiao M. Xu J. Wang W. Zhang B. (2022). Targeting cell death pathways for cancer therapy: recent developments in necroptosis, pyroptosis, ferroptosis, and cuproptosis research. J. Hematol. Oncol. 15, 174. 10.1186/s13045-022-01392-3 36482419 PMC9733270

[B59] Von Krusenstiern A. N. Robson R. N. Qian N. Qiu B. Hu F. Reznik E. (2023). Identification of essential sites of lipid peroxidation in ferroptosis. Nat. Chem. Biol. 19, 719–730. 10.1038/s41589-022-01249-3 36747055 PMC10238648

[B60] Wallin R. Hutson S. M. (2004). Warfarin and the vitamin K-dependent gamma-carboxylation system. Trends Mol. Med. 10, 299–302. 10.1016/j.molmed.2004.05.003 15242675

[B61] Wang L. Fu H. Song L. Wu Z. Yu J. Guo Q. (2022). Overcoming AZD9291 resistance and metastasis of NSCLC via ferroptosis and multitarget interference by nanocatalytic sensitizer plus AHP‐DRI‐12. Small 19, e2204133. 10.1002/smll.202204133 36420659

[B62] Wang R. Hu Q. Wang H. Zhu G. Wang M. Zhang Q. (2021a). Identification of Vitamin K3 and its analogues as covalent inhibitors of SARS-CoV-2 3CL(pro). Int. J. Biol. Macromol. 183, 182–192. 10.1016/j.ijbiomac.2021.04.129 33901557 PMC8064871

[B63] Wang W. Jin Y. Xu Z. Liu X. Bajwa S. Z. Khan W. S. (2020). Stimuli-activatable nanomedicines for chemodynamic therapy of cancer. Wiley Interdiscip. Rev. Nanomed Nanobiotechnol 12, e1614. 10.1002/wnan.1614 32011108

[B64] Wang X. Xu S. Zhang L. Cheng X. Yu H. Bao J. (2021b). Vitamin C induces ferroptosis in anaplastic thyroid cancer cells by ferritinophagy activation. Biochem. Biophys. Res. Commun. 551, 46–53. 10.1016/j.bbrc.2021.02.126 33714759

[B65] Wellington K. W. Hlatshwayo V. Kolesnikova N. I. Saha S. T. Kaur M. Motadi L. R. (2020). Anticancer activities of vitamin K3 analogues. Invest. New Drugs 38, 378–391. 10.1007/s10637-019-00855-8 31701430

[B66] Welsh J. Bak M. J. Narvaez C. J. (2022). New insights into vitamin K biology with relevance to cancer. Trends Mol. Med. 28, 864–881. 10.1016/j.molmed.2022.07.002 36028390 PMC9509427

[B67] White E. (2015). The role for autophagy in cancer. J. Clin. Invest. 125, 42–46. 10.1172/JCI73941 25654549 PMC4382247

[B68] Wu Y. Zhang S. Gong X. Tam S. Xiao D. Liu S. (2020). The epigenetic regulators and metabolic changes in ferroptosis-associated cancer progression. Mol. Cancer 19, 39. 10.1186/s12943-020-01157-x 32103754 PMC7045519

[B69] Xiong Y. Xiao C. Li Z. Yang X. (2021). Engineering nanomedicine for glutathione depletion-augmented cancer therapy. Chem. Soc. Rev. 50, 6013–6041. 10.1039/D0CS00718H 34027953

[B70] Yang G. G. Zhang H. Zhang D. Y. Cao Q. Yang J. Ji L. N. (2018). Cancer-specific chemotherapeutic strategy based on the vitamin K3 mediated ROS regenerative feedback and visualized drug release *in vivo* . Biomaterials 185, 73–85. 10.1016/j.biomaterials.2018.08.065 30227273

[B71] Yang W. s. Sriramaratnam R. Welsch M. e. Shimada K. Skouta R. Viswanathan V. s. (2014). Regulation of ferroptotic cancer cell death by GPX4. Cell 156, 317–331. 10.1016/j.cell.2013.12.010 24439385 PMC4076414

[B72] Yang W. S. Stockwell B. R. (2016). Ferroptosis: death by lipid peroxidation. Trends Cell Biol. 26, 165–176. 10.1016/j.tcb.2015.10.014 26653790 PMC4764384

[B73] Yu B. Choi B. Li W. Kim D.-H. (2020). Magnetic field boosted ferroptosis-like cell death and responsive MRI using hybrid vesicles for cancer immunotherapy. Nat. Commun. 11, 3637. 10.1038/s41467-020-17380-5 32686685 PMC7371635

[B74] Yu P. Zhang X. Liu N. Tang L. Peng C. Chen X. (2021). Pyroptosis: mechanisms and diseases. Signal Transduct. Target. Ther. 6, 128. 10.1038/s41392-021-00507-5 33776057 PMC8005494

[B75] Zhang C. Liu Z. Zhang Y. Ma L. Song E. Song Y. (2020a). Iron free" zinc oxide nanoparticles with ion-leaking properties disrupt intracellular ROS and iron homeostasis to induce ferroptosis. Cell Death Dis. 11, 183. 10.1038/s41419-020-2384-5 32170066 PMC7070056

[B76] Zhang S. Xin W. Anderson G. J. Li R. Gao L. Chen S. (2022a). Double-edge sword roles of iron in driving energy production versus instigating ferroptosis. Cell Death Dis. 13, 40. 10.1038/s41419-021-04490-1 35013137 PMC8748693

[B77] Zhang X. Wu S. Guo C. Guo K. Hu Z. Peng J. (2022b). Vitamin E exerts neuroprotective effects in pentylenetetrazole kindling epilepsy via suppression of ferroptosis. Neurochem. Res. 47, 739–747. 10.1007/s11064-021-03483-y 34779994

[B78] Zhang Z. Ding Y. Li J. Wang L. Xin X. Yan J. (2020b). Versatile iron-vitamin K3 derivative-based nanoscale coordination polymer augments tumor ferroptotic therapy. Nano Res. 14, 2398–2409. 10.1007/s12274-020-3241-7

[B79] Zhou H. Chen J. Fan M. Cai H. Dong Y. Qiu Y. (2023a). KLF14 regulates the growth of hepatocellular carcinoma cells via its modulation of iron homeostasis through the repression of iron-responsive element-binding protein 2. J. Exp. Clin. Cancer Res. 42, 5. 10.1186/s13046-022-02562-4 36600258 PMC9814450

[B80] Zhou M. Yuan M. Jin Y. Zhou Q. Yu Y. Li J. (2023b). Vitamin B2‐based ferroptosis promoter for sono‐enhanced nanocatalytic therapy of triple‐negative breast cancer. Adv. Funct. Mater. 33. 10.1002/adfm.202303899

[B81] Zhu L. You Y. Zhu M. Song Y. Zhang J. Hu J. (2022). Ferritin-hijacking nanoparticles spatiotemporally directing endogenous ferroptosis for synergistic anticancer therapy. Adv. Mater 34, e2207174. 10.1002/adma.202207174 36210735

